# Recovering Genetic Regulatory Networks from Chromatin Immunoprecipitation and Steady-State Microarray Data

**DOI:** 10.1155/2008/248747

**Published:** 2008-06-05

**Authors:** Wentao Zhao, Erchin Serpedin, Edward R Dougherty

**Affiliations:** 1 Electrical and Computer Engineering Department, Texas A&M University, College Station, TX 77843, USA; 2 The Translational Genomics Research Institute (TGen), 400 North Fifth Street, Suite 1600, Phoenix, AZ 85004, USA

## Abstract

Recent advances in high-throughput DNA microarrays and chromatin immunoprecipitation (ChIP) assays have enabled the learning of the structure and functionality of genetic regulatory networks. In light of these heterogeneous data sets, this paper proposes a novel approach for reconstruction of genetic regulatory networks based on the posterior probabilities of gene regulations. Built within the framework of Bayesian statistics and computational Monte Carlo techniques, the proposed approach prevents the dichotomy of classifying gene interactions as either being connected or disconnected, thereby it reduces significantly the inference errors. Simulation results corroborate the superior performance of the proposed approach relative to the existing state-of-the-art algorithms. A genetic regulatory network for *Saccharomyces cerevisiae* is inferred based on the published real data sets, and biological meaningful results are discussed.

## 1. Introduction

Currently, one of the most important research problems in molecular biology and bioinformatics consists of finding out the mechanisms that govern the gene regulations, which are considered to play fundamental roles in the operation of all processes taking place in living cells. Learning the structure and machinery of gene regulations opens up the possibility for understanding and controlling the functioning of organisms at the molecular level, and for designing intelligent therapies and drugs. In a biological process such as cell cycle or environmental response, a gene's product, the protein, can serve as a transcription factor of a target gene by binding to the target gene's regulatory region on chromatin and affect its transcription. The protein can also influence another gene's expression in subsequent stages, for example, through splicing or translation. Alternatively, these protein-gene relationships can be viewed as gene-gene interactions, and are modeled in general as genetic regulatory networks.

Recent years have witnessed a number of different frameworks for modeling genetic regulatory networks, ranging from fine-scale modeling at the molecular level in terms of partial differential equations and stochastic equations, to large scale modeling at the gene and protein-level in terms of Boolean and probabilistic Boolean networks, and (dynamic) Bayesian networks; see, for example, [1–6] and their toolboxes [7–9]. The small scale modeling techniques are used to capture the detailed biochemical aspects of molecular interactions and are in general very computational demanding. On the other side, the large-scale models provide a global vision of the interactions among the constituent elements of genetic regulatory networks and are generally represented in terms of graphs.

In the middle of 1990s, the birth of DNA microarrays equipped the industry with the capability to measure simultaneously the concentration of genome-wide mRNA expressions. The gene expression data produced thereafter by gene chips have attracted extensive research on the inference of genetic regulatory networks based on various network models [10–18]. There are two types of DNA microarray data sets: time series (or time dependent) and time independent (also called steady-state or single point time series) data sets. In general, the time-independent gene expression profiles are capable of recovering steady-state attractors, but fail to recover the direct and oriented (temporal regulating) relationships. On the other side, time series data sets can improve the inference greatly in contrast to time-independent data sets [13]. However, the financial costs, ethnical concerns, and implementation issues prevent collecting beneficial time series data. Recent statistics show that about 70% of published data are time independent [19]. Therefore, the steady-state analysis is highly valuable despite the difficulty of making accurate inference of temporal relationships.

Inference of gene regulatory networks based solely on the information provided by microarray data is limited by a number of factors: number of available microarrays, quality of data samples, experimental noise, and errors (cross-hybridizations). It is also known that post-transcriptional modifications and transcripts that are present at low levels are generally not detectable by microarrays. Since the gene activity is measured by the mRNA level, the underlying assumption is that there is a significant correlation between the mRNA level and the amount of protein associated with mRNA. However, the magnitude of such a correlation varies significantly depending on the type of protein involved. Therefore, a combined approach which, besides gene expression data, exploits additional data sources is likely to enhance the inference process.

The advent of in vivo chromatin immunoprecipitation (ChIP) assays has enabled to test whether a protein acting as a transcription factor binds to a specific DNA segment. Hence, ChIP assays serve as a promising mechanism to examine the regulatory relationships. In ChIP experiments, the protein is immobilized on the chromatin, then the chromatin is broken into DNA fragments, and the DNA-protein complexes are immunoprecipitated by using antibodies corresponding to the tested protein. Afterwards the DNA bound by the protein in question can be isolated and identified by using a cDNA microarray chip. The whole process is also called a ChIP-chip experiment, and inherits several disadvantages. The protein to be tested has to possess a specific antibody, which might not be synthesized, discovered, or exist. In addition, the transcriptional regulation is a complex process that is expressed in several different aspects. The binding of the transcription factor to the promoter region of the target gene is the most pristine mode. Especially for eukaryotic organisms, some regulatory bindings take place at a region far away from the regulated gene. This fact makes the binding information questionable for determining the regulation relationships. Furthermore, the experimental results are represented by p-values and the determination of the binding relationship is achieved through threshold comparison. However, the selection of the p-value threshold introduces a dilemma. A high threshold not only identifies the most probable binding relationships but also might miss many true relationships with lower p-values, while a low threshold infers more relationships, among which more might be false alarms. A good tradeoff is not easy to make. Besides, the cost factor has also to be considered. Generally, ChIP-chip experiments are very expensive and testing thousands of proteins is not affordable.

A combination of both steady-state microarray data and ChIP-chip data might help in making more accurate inferences. Intuitively, these two different types of data complement the shortcomings of each other. This motivates us to propose a Bayesian approach to analyze jointly both data sets and to establish a confidence measure of gene interactions. The proposed scheme possesses six key features which make it different from the existing algorithms. First, gene expression data in steady-state are considered, while time course data are used in other works like [11,13,20]. Second, most of the current schemes recover a unique genetic network represented by a graph which best fits the observed data in a certain metric, while the proposed approach determines the posterior probabilities for all gene-pair interactions and avoids to make a dichotomous decision that classifies each gene interaction as being either connected or disconnected. The proposed approach can be easily transformed into a dichotomous scheme by only preserving the highly probable gene interactions. Third, the underlying structural model is assumed to be a directed cyclic graph, which allows cycles (feedback loops) and directed acyclic graphs are treated as special cases. This contrasts to Bayesian networks, which are directed acyclic graphs. Feedback loops are a common network motif in biological processes and their function is to yield the necessary redundancy and stability for the system [1]. Therefore, methods based on Bayesian networks, for example, [21–23], lose their validity in the inference of cyclic graphs. Fourth, the proposed approach assumes continuous-valued variables, and this prevents the information loss incurred by data quantization. This represents an advantage compared with the discrete-valued networks such as [21–23]. Fifth, the proposed connectivity score is oriented and has a very clear meaning, in the sense of posterior probabilities, while the existing scores based on the mutual information [14,18,24] are vague and lack orientation information. Sixth, in the proposed approach the system kinetics are assumed to be nonlinear, while linear models are commonly utilized for the purpose of simplification [12,15]. Besides, the proposed scheme establishes a general framework whose components can be customized to fit the nature of the underlying biological system.

The rest of the paper is organized as follows. Section 2 discusses the graphical model and system dynamics that govern the genetic expressions. Section 3 translates the p-values of ChIP-chip experiments into regulation probabilities and formulates the inference algorithm through Bayesian analysis. In Section 4, the proposed algorithm and other three schemes are simulated on a set of artificial networks. Performance comparisons illustrate that the proposed algorithm exceeds in terms of several metrics. The robustness of kinetics model is also discussed via simulations. Realistic data sets are exploited in the proposed inference framework and a genetic network is presented to account for the genetic response to environmental changes. Finally, Section 5 concludes the paper with remarks on possible future works.

## 2. Methods

Genetic regulatory networks can be represented by a parameterized graph , where  and  stand for the graph structure and parameter set, respectively. The graph structure qualitatively explains the direct gene interactions, while the parameter set quantitatively describes the system kinetics.

### 2.1. Structural Model

The graph  is employed to map gene interactions at transcriptional level, where  denotes the set of vertices (genes) and  stands for the set of edges (regulation relationships). If gene  regulates gene , graphically such a relation is represented in terms of an oriented edge , where  is a parent of  and  is considered a child of . All genes that present incidence edges with gene  represent the set of parental genes of , and are compactly denoted in terms of the notation . If two genes  and  interact with each other but the regulation orientation cannot be determined, an undirected edge is laid between the two genes as , which means both orientations are possible. A sequence of consecutive-oriented edges constitutes a directed path. If there is no directed path which starts and ends at the same vertex, in other words, the graph contains no loops, the graph is called a directed acyclic graph (DAG). DAGs lie at the basis of Bayesian networks, which are commonly employed to model causal relationships [25].

General directed graphs (with possibly cycles) will serve as our structural model since they are consistent with the features exhibited by biological systems, in which loops account for system redundancy and stability. Given the graph structure , the parent set  is specified for any gene . For conciseness, the subscript  associated with some variables is omitted in the analysis procedure when the context has clearly specified the gene in question. Next, we discuss the system kinetics and parameters defined in .

### 2.2. System Kinetics

The system kinetics represents the dynamics that governs the gene mRNA concentrations in terms of gene-gene interactions. It can be modeled by a set of differential equations (DEs). A simplified form is a set of linear DEs. However, we accept the more complex model which was employed previously by [16,17] since it is much more realistic and accounts for the expression saturation. Given a gene , its parent set  can be further partitioned into two disjoint subsets: the activator set  and the repressor set , that is,  and . The kinetics of gene  can be explained by the following differential equation:(1)

where  is the concentration of gene 's transcriptional product, namely, mRNA. In this paper, to simplify the exposition, the gene name and its expression are used interchangeably. The changing rate of gene  is controlled by its activating and repressing parents, denoted individually by  and .  and  serve as the regulating factors corresponding to each activator and repressor.  and  assume positive values, and hence can be modeled by a gamma distribution with shape and scale parameters . Here we can unbiasedly assume that the activators and repressers share the same gamma distribution for their regulation factors. Other light-tail distributions, such as Weibull and lognormal distributions, could also be employed. However, since gamma distribution is popular in modeling the reaction rate or molecular concentration [26], the gamma distribution is chosen here.  stands for the gene degradation rate and the time scale can be properly chosen in order to normalize  to the unit value .  represents the expression baseline rate, taht is, the expression rate for  when there is neither activator nor repressor regulating the target gene . Suppose that  represents the observation of , then  assumes the form , where  incorporates all noise sources and is modeled by an additive Gaussian random variable with zero mean and variance .

As the response to environmental changes or incitations, a mature biological system always converges to a certain steady-state, in which all genes stay in equilibrium and do not change their expressions. In this context, the periodic processes, for example, cell cycle and circadian rhythm, are excluded from our research interest. By setting  and , the observation  of the steady-state gene expression for gene  can be expressed as(2)

Given a parent structure  for gene , the parameters in  can be summarized as follows.

(1) For each parent , a binary variable is demanded to specify whether the parent is an activator or repressor, that is, , where  is the indicator function and it assumes the value 1 when , and 0 otherwise. It can be modeled by a Bernoulli random variable with known success probability .

(2) For each activator  and repressor , it is assumed that the regulating factors , where  are known.

(3) The baseline parameter  is usually known.

(4) The noise , where  can be set to a specific value or estimated.

It is worth to note that the choice of nonlinear differential equation and parameter priors does not influence the flow of analysis. Our scheme stands for a general framework and the detailed parameters can be easily customized to fit different scenarios. There are various mathematical models for system kinetics, such as [27–29]. The kinetics in 1 is chosen as our dynamic model because it possess the property of saturation, a key idea of Michaelis-Menten kinetics [29]. Besides, it is fairly simple and it also takes account of most other biological properties. Therefore, in the simulation of the real data set, we are assuming the proposed kinetics is true.

## 3. Inference Method

Consider a system composed of  genes indexed by . ChIP-chip experiments can be conducted to examine whether gene 's corresponding protein binds gene 's regulatory region. Usually this regulatory sequence is a promoter region which is located within 600 base pairs upstream of the coding region of gene . The experimental results are represented in terms of p-values. In the first step, it is necessary to translate the p-value  into the probability of existence of a regulation relationship from gene  to gene , which is denoted as . This probability will act as the prior knowledge to integrate gene expression data.

### 3.1. Incorporating ChIP-Chip Data

The p-value is within the range of . After studying the properties of the microarray data, Allison proposed to exploit mixed Beta distribution to model the p-value [30]. If the transcription factor  regulates gene , it is assumed that the ChIP-chip experiment produces a p-value  which conforms to a Beta distribution with parameters ,(3)

where  stands for the probability density function and  represents the beta function. On the other hand, if  does not regulate , the p-value assumes a different Beta distribution with parameters :(4)

Based on the knowledge provided by established and verified genetic networks, one can infer a prior knowledge about the probability of connectivity between arbitrary genes, denoted as  for all . Such statistics regarding the network connectivity can be found in the open literature, for example, the data sets for yeast [31], and Drosophila [32]. By applying Bayes theorem, we obtain(5)

For simplicity, a uniform distribution can be alternatively employed to account for the p-value when . In this case, , and (5) takes the form(6)

The determination of  and  depends on the experimental knowledge of the accuracy of selecting p-value thresholds. In the first step, a p-value threshold  is imposed, then the validity of all bindings with p-values less than  is corroborated by biological experiments. In this way, we can gain knowledge of the probability , which can be written in the form of(7)

Some works in the literature, for example, [33], have made the observation that at a p-value threshold of 0.001, the frequency of false positives is 6%–10%, that is, . Taking into account these special points, we can determine the pair  in a small range. In our case,  and . Finally, a table can be set up to map the p-value into the edge existence probability, which can be computed only once. It is an overhead for the computational system but it does not assume much computational resource in the runtime.

### 3.2. Exploiting Steady-State Gene Expression Data

Assume that  observations of expression vector are obtained and stored in matrix . Next, we develop a computational approach to establish the posterior probability of the regulation , that is, the probability of the existence of the edge , which is represented by . This posterior can be obtained through integration over the whole parental gene set and parameter space for gene :(8)

where the function  is the indicator function, which takes 1 if  and 0 otherwise. Applying Bayes theorem,  can be expressed as(9)

where  denotes the observations of gene , and  represents the collection of all the observations pertaining to all genes excluding those of gene .  denotes the probability density of the high-dimensional parental model given the observation of ChIP-chip data.  stands for the gene expression likelihood given the parental values and the graphical model. It is a Gaussian distribution with known variance and mean determined by the first part of (2). The second equality in (9) holds because we believe the ChIP-chip experiment and steady-state gene expression measurements are independent. By plugging (9) into (8), it can be inferred that(10)

The integrations at the numerator and denominator of (10) cannot be generally performed in a closed-form expression. However, the Monte Carlo methods enable to numerically evaluate the posterior probabilities. We can generate Monte Carlo samples based on the model probability density  and the integration can be obtained by averaging over these samples. Then the posterior probabilities can be estimated by(11)

Assuming that the selection of a parent as an activator is performed in an independent manner, and that the selection of the regulation factor value is also performed independently, the model probability density  can be further expanded by using the chain rule(12)

Equation (12) conveys the idea that the random samples of graphical models can be sequentially created and processed. First the network structure is created based on the binding probability of gene regulation obtained in the ChIP-chip experiment, then each parent is randomly assigned to represent an activator or repressor, and finally regulation factors are generated.

### 3.3. Algorithm Formulation

Our computational procedure can be briefly formulated in terms of Algorithm 1, where the Matlab coding convention is used to write the pseudocode. There exist  genes in the system. An  matrix is created to represent the p-values produced in the ChIP-chip experiment. We collect  steady-state gene expression samples. The output entry  stands for , and  denotes the number of Monte-Carlo iterations. Lines 1 and 2 deal with the ChIP-chip experimental data and translate p-values into the binding probabilities by using (5). The results are stored in matrix . Lines 3 and 4 perform the preprocessing of the gene expression data. Let  be the values of a specific gene expression in ascending order. The smallest two values, , and the largest two values, , are treated as outliers and discarded. The dynamic range is defined as . The gene expressions are normalized as follows: the smallest two samples are assigned the null value and the largest two samples are assigned the unit value; the intermediary samples  are normalized as ; if there is a missing sample, it is recovered through interpolation by gene's mean expression. Lines 12 through 16 implement the numerator of (11), and Line 17 computes the denominator of (11).

The algorithm can be easily reorganized into a parallel form so that we can exploit efficiently the distributed computational resources. The entries of output matrix  represent the posterior probabilities of regulation relationships between any two genes. It is directional (asymmetrical), and it possesses a clear probabilistic meaning compared with other vague connectivity metrics, for example, mutual information. It grants the biologists the flexibility first to examine the most significant interactions, then to proceed with less evidenced edges. Therefore, it is advantageous relative to a purely dichotomous scheme, in which genes are treated as being either connected or disconnected. A probability threshold can be imposed to change the algorithm into a dichotomous classifier. Since the posterior probability has a universal meaning, this threshold can be easily selected, usually within the range of [0.3–0.9]. A tradeoff has also to be made for different performance metrics.

**Algorithm 1:** Inference of connectivity significance.

(1) Input ChIP-chip data set ;

(2) Translate p-values to construct the binding probability matrix .

(3) Input gene expression data set ;

(4) Normalize the expression data so that each expression is within the range

        of ;

(5) Initialize ;

(6) *for* to *do*

(7) * *Randomly create a directed graph and the adjacency matrix  based on

* *        ;

(8) * for* to *do*

(9) * *    For gene 's parents specified in , randomly assign them to be

        * *    activators or repressers;

(10) * *    For each parent, randomly create their regulation factor  or ;

(11) * *    ;

(12) * *    *for* to *do*

(13) * *       *if**then*

(14) * *         ;

(15) * *       *end if*

(16) * *     *end for*

(17) * *     ;

(18) * end for*

(19) *end for*

(20) ;

(21) *return*.

## 4. Results

The simulation consists of two parts. In the first part, artificial networks are created and the performance of the proposed algorithm is compared with other representative algorithms available in the literature, namely the relevance network (RN) method [14], Chow-Liu algorithm [24], and ARACNE [18]. In the second part, the algorithm is tested on the real *Saccharomyces cerevisiae* (budding yeast) data set and a biologically meaningful genetic network is inferred for the genetic response to environmental changes.

### 4.1. Simulation on Artificial Networks

The proposed algorithm is compared with other three algorithms to evaluate its capability of recovering genetic networks based on gene expression data alone. The relevance network (RN) model [14] represents a robust inference method based on gene expression profiles. In the first step, it computes the mutual information between any two genes  and , denoted as . Then it suggests two genes  and  to be relevant if their mutual information assumes a larger value than a prespecified threshold and it lays down an undirected edge as . Hence, RN measures the significance of gene interactions in terms of mutual information between the gene expressions and produces an undirected cyclic graph. Chow-Liu algorithm [24] approaches the inference problem by finding the maximum spanning tree in which the edge weights stand for the mutual information. However, it loses validity if the underlying model is a cyclic graph. In addition, when the graph is densely connected, this scheme might falsely miss too many edges. ARACNE algorithm [18] exploits the data processing inequality (DPI). It starts with a fully connected graph and a predefined mutual information threshold. Whenever the mutual information between two genes  and , that is, , is less than a threshold, it disconnects the two genes. Next, in the preliminary graph if there exists  so that , then it disconnects  and . In our simulations, we resort to an already available but efficient Matlab toolbox [34] to estimate the mutual information.

#### 4.1.1. Performance Definition

Before making performance comparisons, we define inference errors and performance metrics. Because RN, Chow-Liu, and ARACNE algorithms all construct undirected graphs, we have to disregard the orientation information inferred by the proposed algorithm. The synthetic and inferred graphs are represented by  and , respectively. The two graphs share the same set of vertices but differ in the set of edges.

There are two types of inference errors. The type-1 errors are false positives (FP) and are also called false alarms. If the inference algorithm determines an interaction for two vertices  and  in the inferred graph, denoted as , but there is no such edge in the synthetic graph, that is, , then an FP is produced. The number of FPs, represented by , can be counted as follows:(13)

where  stands for the logic and operator. The type-2 errors are false negatives (FN) and also named misses. If the inference does not discover the connectivity  which resides in the synthetic network, an FN is generated. The number of FNs, depicted by , is obtained by(14)

Correct inference can also be divided into two categories. If  and , the correctness is defined as a true positive (TP). Its summation, annotated by , is(15)

On the other hand, if  and , this correctness is called a true negative (TN). The number of TNs, represented by , is defined as follows:(16)

Different performance metrics are proposed in the literature. Three most popular of them are considered here. The first metric, referred to as the Hamming distance, is the summation of all the inference errors and is given by(17)

The Hamming distance is widely accepted as a good measure of the distance between two graphs.

The second metric is called the sensitivity, and is defined as(18)

The sensitivity describes the inference algorithm ability to identify the regulation relationships among genes. The third metric is called the specificity, and it assumes the form(19)

The specificity represents the inference algorithm's capability to avoid falsely connecting two unrelated genes.

#### 4.1.2. Simulation on the Proposed Kinetics

A set of artificial networks are created based on the system dynamic equation (1). Each network has 30 vertices and 60 oriented edges. Such a network scale is selected for the consideration of the computational resources and the biological network that we are going to infer. The steady-state data are sampled by emulating the gene knockout experiment. A gene's expression is mandatorily forced to 0 while all other genes are free to change their expressions. The initial values of the system are randomly generated. When the system converges to the equilibrium, a Gaussian noise  is added and a few samples are obtained. All genes are shut down one by one. An extra in silico experiment is performed and no genes are shut down. These samples correspond to the wild type strain.

Different numbers of steady-state samples were generated based on the adopted system kinetics. The transcription factor is assumed to be an activator or repressor with equal probability, that is, . The baseline parameter  and the gamma parameters of regulation factors are  so that the regulation factor has a unit mean. Chow-Liu algorithm creates a spanning tree; therefore, it preserves only 29 edges, while the original synthetic network possesses 30 vertices and 60 edges. In order to make comparisons, we tune the parameters for the other three schemes so that the number of inferred edges is around 30. For the RN method, we keep the 30 edges with the highest mutual information. For ARACNE, the mutual information threshold is adjusted. In our proposed algorithm, the posterior probability thresholds are changed in the range of  so that approximately 30 edges are obtained. It has to be noted that RN, ARACNE, and Chow-Liu algorithms only preserve interactions but disregard the interaction orientation. Therefore, in order to make consistent comparisons, we have to sacrifice the orientation information offered by the proposed algorithm. Besides, these three schemes have no capability of processing ChIP-chip data. Therefore, we have to configure the proposed algorithm such that any two nodes are associated with a small prior probability of connection (0.1). This reflects the fact that the connection between two arbitrary nodes in the graph is very unlikely, but not impossible. This also exemplifies how the algorithm works in the absence of the ChIP-chip data.

**Figure 1:**
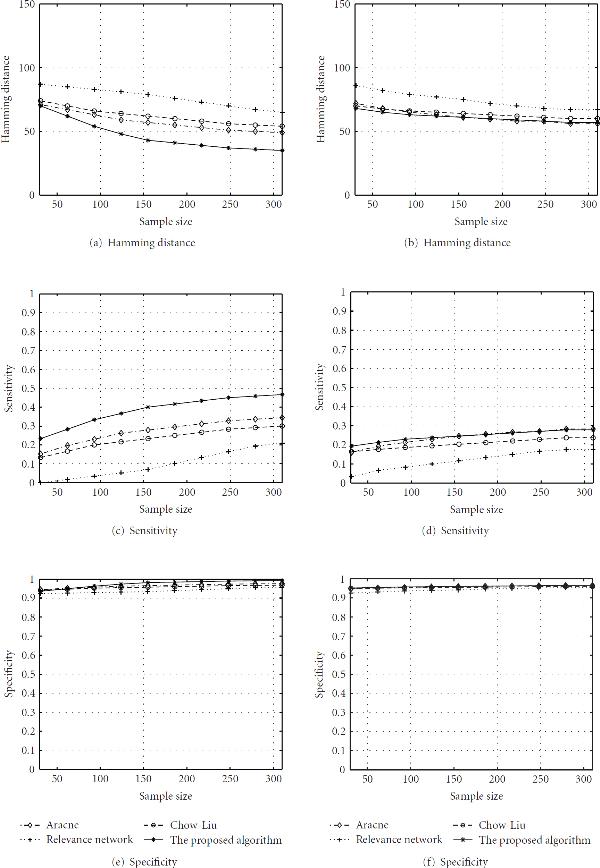
**Performance comparison in terms of Hamming distance, sensitivity and specificity**. Figures in the left column illustrate results based on the same kinetics model employed in both data synthesization and network inference, while figures in the right column represent results based on different kinetics models employed in the simulation process. The Monte Carlo iterations are fixed at  for the proposed algorithm. Thresholds for different algorithms are selected to produce around 30 inferred edges.

Figure 1(a) compares the performance in terms of Hamming distance for the four schemes assuming different sample sizes. The proposed method provides much better inference accuracy because it achieves the lowest Hamming distance. Larger sample size rewards a better inference precision. Chow-Liu's algorithm and ARACNE do not perform well. This can be attributed to the assumption of the network. Our synthetic networks actually are cyclic networks in order to reflect the real world scenario. However, cycles in the network ruin the inference precisions of Chow-Liu and ARACNE. Figure 1(c) illustrates the impact of sample size on the sensitivity. The proposed scheme outperforms the other three schemes. The sensitivities of all algorithms are less than 0.5. This is mainly due to the constraint that we pose on the number of inferred edges, that is, 30 edges. If we relax the posterior probability threshold, the sensitivity will be improved by sacrificing the specificity. Figure 1(e) depicts specificity for all schemes. All of them have high specificities, which are all greater than 0.90. The proposed scheme still exceeds. This high specificity is mainly due to the stringent constraint posed on the number of inferred edges. When considering the orientation of the edges, we find that 90% true positives inferred by the proposed algorithm are actually oriented correctly. This represents a big advantage of the proposed algorithm compared with the other three schemes.

#### 4.1.3. Robustness of Inference

In the previous simulations, the proposed inference algorithm assumes the system dynamic as depicted by (1). Actually, for different biological processes, there exist various mathematical models which achieve tradeoffs between the sophistication of the underlying molecular reaction and the simplification of the formula description (see [27,29] for model comparisons). Savageau [28] proposed an alternative mathematical model to account for the gene control and various forms of coupling among elementary gene circuits. This model can be denoted as(20)

where two new symbols  and  are activation and degradation coefficients and all other symbols share the same meanings as in (1).

Although the proposed inference framework can "plug and play" with different models, it is still necessary to examine its robustness against the underlying model. We evaluate this model dependence by the following steps: configure the model as 13 and create a set of synthetic data, then apply the proposed algorithm based on the dynamic equation (1), finally determine the performance metrics for different algorithms and compare the results with those in the previous section.

The simulation results are plotted in Figures 1(b), 1(d), and 1(f). Each figure corresponds to a different performance metric. All algorithms exhibit different values for performance values. This shows that the inference is dependent on the particular data sets and their underlying model. Compared with other three schemes, the proposed algorithm still achieves good performance in terms of three metrics. However, the advantage of the proposed algorithm are not significant now. ARACNE, Chow-Liu, and relevance method do not degenerate much. This attributes mainly to the nonparametric property of these three schemes. The persistent good performance of the proposed algorithm is due to the fact that both dynamic models have to convey the basic properties of the gene interaction kinetics, such as the activation and repression effects and the coupling of the circuitry.

### 4.2. Simulation on Saccharomyces Cerevisiae Data Sets

*Saccharomyces cerevisiae* (yeast) has been extensively studied in the literature of molecular biology because it is a unicellular eukaryotic organism, which shares similar cell structure with plants and animals. Also, yeast presents a short life cycle, which makes the experiments to be easily conducted. Lee et al. [33] performed the ChIP-chip experiment, in which 141 transcription factors were tested for binding intergenetic regions corresponding to 6270 genes. The gene expression data were published by Mnaimneh et al. [35], who created promoter shut-off strains for 2/3 of all essential genes. The data set contains 215 steady-state cDNA microarray samples. The model parameters are assumed the same as artificial networks.

**Figure 2:**
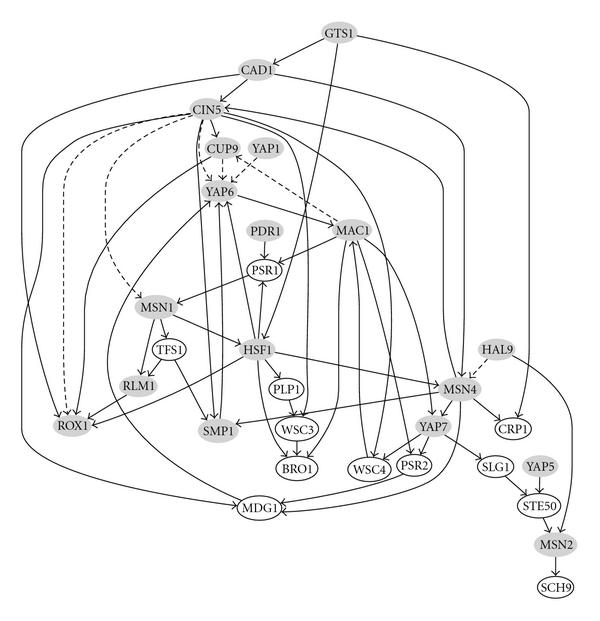
**Recovered genetic regulatory network for yeast stress response**. The Monte Carlo iterations are . Dashed edges represent interactions preserved by using ChIP-chip data alone under the p-value threshold 0.001. Shadowed vertices are transcription factors tested in the ChIP-chip experiment.

The intracellular signalling pathway in response to environmental changes has been conserved through evolution. Therefore, a study of this biological subsystem on the *Saccharomyces cerevisiae* might help to decipher the cell survival mechanism of other organisms. We select 30 genes which are annotated to participate in the stress response process. The given ChIP-chip experiment did not provide full prior knowledge between any two genes (nodes in the graph). We believe that, among these genes, there are some genes whose protein products may also serve as transcription factors. Therefore, if the binding between two genes was not tested in the ChIP-chip experiment, a small probability value 0.1 is assigned as the prior knowledge. The proposed inference algorithm leads to the genetic network illustrated in Figure 2.

The inferred genetic regulatory network shows strong proneness toward a scale-free network instead of a random network. Some genes possess especially high degree of connectivity. Three hub genes  already connect with more than 60% of all selected genes. Each of them has a connectivity degree not less than 8 while on average each gene in the network is connected with no more than 4 genes. These hub genes constitute the backbone of the network and they are potential control targets. This scale-free property is advantageous in maintaining the system robustness because a failure in one subsystem will not be propagated to the whole body.

Multiple works, for example [36], have identified  and  as two of the most important genes in the response to environmental changes. A recent work [37] recognized the functionality of another crucial gene , which is a heat shock transcription factor and functions in a different domain than the one corresponding to . Our inferred network confers this experimental result by showing that  and  regulate different set of genes except a weak connectivity between  and .  are not conserved in humans, while  genes have been preserved for various organisms such as Drosophila melanogaster, chickens, and mammals. Therefore, a study of the  pathway opens up the possibility of understanding the mechanism that governs the survival of normal cells under austere conditions.

 () and  are two genes that play key roles in controlling the resistance to drugs, for example, cisplatin [38]. ,, and  share a structure motif called basic leucine zipper () and they are located closely in the network. However, they are not neighboring the other two  genes:  and . It is hypothesized that although they have similar molecular structures, their biological functionalities are in distinct domains.

Several edges, discovered by imposing a stringent p-value threshold 0.001 to the location data, were persevered in our inferred network. Actually, these connections constitute a small portion of the proposed network, and they are , and . Various evidences are found to corroborate the recovered interactions, which can not be obtained by employing a stringent p-value for the location data. For example,  is recovered to directly regulate . This regulation relationship has also been reported in the work of Horak [39]. The relationship between  and  is studied in [40] in the context of extending the life span.

It is worthwhile to note that gene expression data mainly provide statistical relationships among genes, while location data offer physical binding interactions at the molecular level. By combining the two data sources, we are aiming to refine the inferred network to be biologically more meaningful. However, it also runs at a risk of confusing statistical regulatory relationships with real binding interactions. When such a case occurs, the proposed algorithm is capable of constraining the interacting genes within the same biological process and common functional relationships. A related discussion about the meaning of inferred network can also be found in [41].

## 5. Conclusions

A novel algorithm is proposed to recover the genetic regulatory networks in the light of knowledge of transcriptional kinetics, ChIP-chip, and gene microarray data. The analysis is based on the Bayesian methodology and Monte Carlo techniques. The proposed scheme is useful to compensate the shortcomings of the utilization of only one data set alone. Our in silico experiments corroborate that the algorithm outperforms in specificity, sensitivity and Hamming distance relative to three state-of-the-art schemes. A budding yeast genetic regulatory network is proposed to account for the stress response.

There are possible extensions to our current scheme. An analysis of the error estimation is desired for the Monte Carlo simulation in order to determine the appropriate number of iterations. Several other knowledge sources are to be integrated into the current framework. For example protein-protein interactions are useful to identify cobinding regulations. Genome sequencing data have been utilized to find regulatory motifs. Protein structure knowledge can be exploited to categorize the proteins and find similar functionality. A cross-species research is also highly desirable since similar regulation mechanisms are expected to be conserved. If a gene is conserved in both humans and mice, then the knowledge of the gene pathway in the mouse will be an excellent reference for the study of human genetic diseases. We expect a global distributed framework, in which each data source acts as a separate component and its absence does not interfere with the whole computational process.
